# Lexical neighborhood effects in pseudoword spelling

**DOI:** 10.3389/fpsyg.2013.00862

**Published:** 2013-11-28

**Authors:** Marie-Josèphe Tainturier, Marie-Line Bosse, Daniel J. Roberts, Sylviane Valdois, Brenda Rapp

**Affiliations:** ^1^School of Psychology, Bangor UniversityBangor, Gwynedd, UK; ^2^Laboratoire de Psychologie et NeuroCognition, CNRS UMR 5105, University of GrenobleFrance; ^3^Cognitive Science Department, Johns Hopkins UniversityBaltimore, MA, USA

**Keywords:** pseudoword spelling, skilled spelling, literacy, spelling models, neighborhood activation

## Abstract

The general aim of this study is to contribute to a better understanding of the cognitive processes that underpin skilled adult spelling. More specifically, it investigates the influence of lexical neighbors on pseudo-word spelling with the goal of providing a more detailed account of the interaction between lexical and sublexical sources of knowledge in spelling. In prior research examining this topic, adult participants typically heard lists composed of both words and pseudo-words and had to make a lexical decision to each stimulus before writing the pseudo-words. However, these priming paradigms are susceptible to strategic influence and may therefore not give a clear picture of the processes normally engaged in spelling unfamiliar words. In our two Experiments involving 71 French-speaking literate adults, only pseudo-words were presented which participants were simply requested to write to dictation using the first spelling that came to mind. Unbeknownst to participants, pseudo-words varied according to whether they did or did not have a phonological word neighbor. Results revealed that low-probability phoneme/grapheme mappings (e.g., /o/ -> aud in French) were used significantly more often in spelling pseudo-words with a close phonological lexical neighbor with that spelling (e.g., /krepo/ derived from “crapaud,” /krapo/) than in spelling pseudo-words with no close neighbors (e.g., /frøpo/). In addition, the strength of this lexical influence increased with the lexical frequency of the word neighbors as well as with their degree of phonetic overlap with the pseudo-word targets. These results indicate that information from lexical and sublexical processes is integrated in the course of spelling, and a specific theoretical account as to how such integration may occur is introduced.

## INTRODUCTION

**FIGURE 1:**
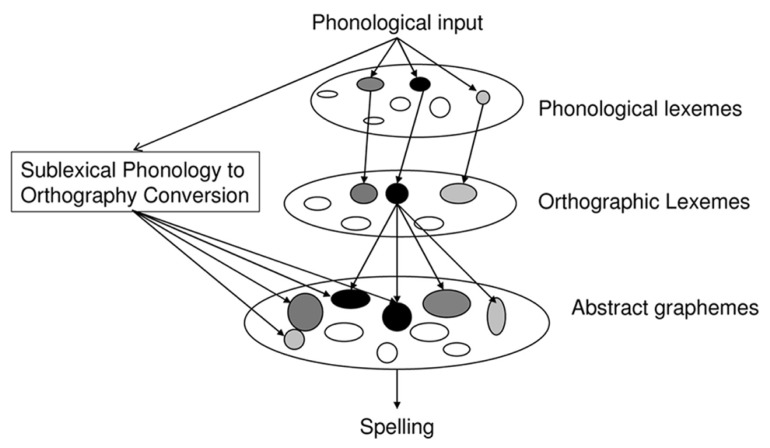
**A functional architecture of spelling to dictation.** Lexical and sublexical activations are integrated at the abstract grapheme level.

Spelling is generally assumed to involve two major processes, or “routes” (**Figure 1**). First, one can access the stored spellings of familiar words in an “orthographic lexicon,” following activation from the phonological lexicon and/or the semantic system. This lexical process is necessary when spelling words with ambiguous or irregular spellings such as “two” or “colonel.” Second, spelling can occur via a sublexical phonology to orthography conversion process. In contrast to the lexical process, the sublexical process can generate plausible spellings for unfamiliar words or pseudo-words. In “deep” orthographies such as English or French, phonemes can often be spelled in several ways. The sublexical spelling process is thought to be sensitive to the relative probability of use of different phoneme-grapheme mappings. For instance, KEET would be more likely than KEIT in response to /ki:t/ because /i:/->EE (as in “meet”) is a more probable mapping than /i:/->EI (as in “seize”; Hanna et al., 1966; Baxter and Warrington, 1987; Barry and Seymour, 1988; Sanders and Caramazza, 1990; Fry, 2004). Thus, spelling words through the sublexical process alone may lead to phonologically plausible errors such as “phone”-> FONE or “colonel” -> KERNEL in which low probability phoneme-grapheme mappings are replaced with higher probability mappings. Indeed, such errors are a characteristic feature of the spelling performance of brain damaged individuals with an impaired lexical process, as observed in “surface dysgraphia” (Tainturier and Rapp, 2001). Although the existence of two processes with these general characteristics has been assumed in most written language research (for a review see: Tainturier and Rapp, 2001) there is little consensus concerning the specific nature of these processes or their relationships. In this paper, we will address the following question: are lexical and sublexical spelling processes essentially independent or do they interact and, if so, how?

Several strands of evidence point to some degree of interaction between lexical and sublexical spelling processes. First, it appears that spelling real words can be influenced by sublexical information. One important source of evidence in this respect comes from the analysis of the spelling performance of dysgraphic patients with an impaired lexical spelling process (e.g., Hillis and Caramazza, 1991; Miceli et al., 1994, 1999; Hillis et al., 1999; Folk et al., 2002; Rapp et al., 2002; Folk and Jones, 2004; Laiacona et al., 2009). For example, the dysgraphia of case LAT was characterized by the production of phonologically plausible errors (Rapp et al., 2002), pointing to a failure of the lexical process with an increased reliance on sublexical conversion when spelling words. However, LAT’s phonologically plausible errors often contained lexically correct elements that were of such low phoneme-grapheme probability that it was very unlikely that they could have been generated by the sublexical process alone (e.g., “bouquet” -> BOUKET; “knowledge” -> KNOLIGE). In support for a lexical source of these elements, LAT produced significantly more low probability spellings (e.g., /eI/ -> ET) in his phonologically plausible, yet erroneous, responses to words than in his spelling of phonologically similar pseudo-words (e.g., spelling /b ou k ei/ -> BOUKET, but /l ou k ei/ -> LOKAY). This indicates that many of LAT’s phonologically plausible errors resulted from the blending of partial lexical knowledge with sublexical information. It has also been shown that word and pseudo-word spelling of dysgraphic patients is influenced by orthographic neighborhood (Sage and Ellis, 2006), which could be taken to imply lexical/sublexical interactivity.

Second, evidence for the interaction of lexical and sublexical information comes from studies of lexical priming effects on pseudo-word spelling in unimpaired adults (Campbell, 1983; Barry and Seymour, 1988; Seymour and Dargie, 1990; Dixon and Kaminska, 1994; Barry and De Bastiani, 1997; Perry, 2003; Folk and Rapp, 2004; Bonin et al., 2005; Martin and Barry, 2012). Typically, participants heard lists composed of both words and pseudo-words and had to make a lexical decision to each stimulus but write down only the pseudo-words. Results showed that the spelling of a pseudo-word could be affected by the orthography of a previously heard word. For example, /pri:t/ is more likely to be spelled PREET following the spoken word “sweet” and to be spelled PREAT following “heat.” This suggests that the orthographic choices of unimpaired adults when spelling pseudo-words to dictation are not merely a function of phoneme-grapheme probabilities but also reflect some lexical influence. However, a limitation of such priming studies is that the tasks (e.g., lexical decision) might have triggered specific strategies that do not reflect processes that are normally engaged when people spell unfamiliar stimuli. This may have occurred because the tasks themselves invoked lexical activation and because in many, though not all, previous studies there was a very obvious relation between word primes and pseudo-word targets. In addition, it has been argued (e.g., Perry, 2003) that priming effects on pseudo-word spelling may reflect a re-weighting of phoneme-grapheme mappings rather than a lexical influence on pseudo-word spelling *per se*.

Finally, supporting evidence for an influence of lexical orthographic knowledge on pseudo-word spelling comes from acquisition studies that have used more naturalistic paradigms less susceptible to strategic effects (Bosse et al., 2003; Martinet et al., 2004). These studies indicate that spelling pseudo-words by analogy to known words may even be the dominant strategy in the early stages of spelling acquisition, before children have developed a sufficiently large word base to generalize phonology to orthography correspondences. However, it remains to be seen whether or not such effects are replicable in adult populations using similar non-strategic paradigms (details below). This is not a trivial question as spelling processes are likely to be affected by the size and variety of the spelling knowledge available at different stages of acquisition. For example, words that include low probability mappings tend to have a very high frequency of use in the language (e.g., “two,” “woman”) and will thus be over-represented in the developing lexicon.

Aside from methodological considerations, the specific mechanisms underlying the lexical influence on pseudo-word spelling remain poorly understood. Some researchers (e.g., Campbell, 1983; Graham et al., 2000; Jefferies et al., 2007) have argued against the distinction between lexical and sublexical processes altogether and have proposed that pseudo-word spelling occurs entirely via a unified lexical analogy process. Others (e.g., Perry, 2003) have proposed that lexical priming can modify the relative weights of competing sublexical phoneme-grapheme mappings. Finally, some authors (e.g., Barry, 1988; Kreiner and Gough, 1990) have made the general suggestion that lexical and sublexical processes, although not directly influencing each other, may interact at an output level. A more specific proposal for a mechanism of lexical/sublexical integration at the output level has been put forward by Rapp et al., (2002; see also: Bosse et al., 2003; Houghton and Zorzi, 2003; McCloskey et al., 2006; Jones et al., 2009; see also Martin and Barry, 2012 for a similar proposal aimed at explaining priming effects). This proposal is based on the notion that there is a level of representation at which graphemic units (letters and/or graphemes and/or orthographic syllables, Tainturier and Rapp, 2003, 2004) are represented and independently activated by the orthographic lexicon, by sublexical phonology to orthography conversion, or both (**Figure 1**). These graphemic representations are maintained active (graphemic buffering) while awaiting production as letter shapes or letter names. In this framework, the selection of a letter string for output results from the integration of these two different sources of activation. This proposal reduces the degree of autonomy of lexical and sublexical processes because both processes activate a common level of graphemic representation. That is, the spelling of either words or pseudo-words is under the combined influence of lexical and sublexical processes.

The goal of the work reported here was twofold. The first was to examine the existence of lexical neighborhood effects on pseudo-word spelling in skilled adult spellers using a paradigm designed to minimize overt or potentially task specific lexical influence and previously used with children (Bosse et al., 2003).

In the work we report here, carried out in French, only pseudo-words were presented to participants who were simply requested to write them to dictation using the first spelling that came to mind. Unbeknownst to the participants, the pseudo-word stimuli varied according to whether or not they had a phonologically close word neighbor with a low-probability spelling. Given that spoken pseudo-words can activate their phonological word neighbors which can in turn activate their spellings (see Discussion), direct evidence for a lexical influence on pseudo-word spelling would be obtained if low-probability spellings (e.g., /i/ -> IT, in French) were used more often when spelling pseudo-words with a phonological neighbor containing the low-probability spelling (e.g., /bəti/ derived from “petit” (small), /pəti/) than when spelling pseudo-words without close lexical neighbors (e.g., /tãzi/).

The second goal of this study was to investigate factors that may modulate the magnitude of the lexical influence on pseudo-word spelling in order to contribute to a more detailed understanding of the specific mechanisms that underpin the interaction between lexical and sublexical processes in the course of spelling. Specifically, we considered the effect of the extent of phonological similarity between a pseudo-word and its lexical neighbor, as well as the effect of the lexical frequency of the neighbors.

## EXPERIMENT 1: LEXICAL INFLUENCE ON PSEUDO-WORD SPELLING

### METHODS

#### Participants

Twenty-nine undergraduate students of the University Pierre Mendès-France (Grenoble) participated in this experiment in exchange for course credits. All were native French speakers and reported no history of neurological disorders or dyslexia.

#### Materials

A list of 76 stimuli was constructed which included three sets of 14 experimental and 34 filler pseudo-words. Experimental pseudo-words were derived from 14 French disyllabic words (8 CV-CV, 2 CVC-CV, 2 CCV-CV, 2 CCV-CV) of medium to high frequency (mean = 204 per million, range: 7–1539; from Lexique 3, New, 2006). These source words all ended with a fairly low probability final phoneme-grapheme correspondence, that is, their spoken forms ended with phonemes which are more typically spelled differently [e.g., /i/-> IT as in /pəti/ -> “petit” (small) rather than the more typical /i/ -> I as in “joli” (pretty), or /o/->OP as in /siro/ -> “sirop” (syrup) rather than the more typical /o/ -> EAU as in “bateau”]. Words with vowel endings were selected because this is where most spelling ambiguities occur in French. Although none of these 14 target graphemes corresponded to the highest probability mapping, they covered a range of probabilities. For instance, three different target graphemes were used for the final phoneme /a/: AT, AS, and AC with PG probabilities of 29.9, 3.6, and 1% respectively. For this phoneme, the most common written correspondence is A which therefore was not selected as a target grapheme as its use would not point to a lexical influence on pseudo-word spelling.

Three sets of pseudo-words were derived from the 14 source words by substituting one or more of their constituent phonemes other than the final target phoneme. Set one included 14 *neighbor pseudo-words*, created by substituting one phoneme only in the first syllable of each source word (e.g., /bəti/ derived from “petit”/pəti/). Importantly, these neighbor pseudo-words were constructed so as to ensure that each one had no close word neighbor other than the source word. Neighbors were operationally defined as words of the same length sharing more than 50% phonemes, in the same position, with the pseudo-word. Thus, neighbor pseudo-words all had one and only one close neighbor, the source word. This ensured that the expected effect of source words on pseudo-word spelling would not be affected, or even masked, by the influence of other close word neighbors with alternate spellings. Set two consisted of 14 *phoneme control pseudo-words*, created by substituting additional phonemes so that the resulting pseudo-words ended with the same final phoneme as the source words but had no close word neighbors (e.g., /tã∞i/ derived from “petit”/pəti/). Set three included 14 *syllable control pseudo-words* which also had no close neighbors but in which the entire final syllable of the source word (rather than only the final phoneme as in Set 2) was preserved (e.g., /liti/ derived from “petit”/pəti/). Finally, filler pseudo-words without neighbors were introduced to keep the proportion of neighbor pseudo-words under 20% and to vary spelling patterns. None of these included the same final phoneme as any of the experimental pseudo-words.

Assuming that the auditory presentation of a pseudo-word results in the activation of close lexical neighbors (see Discussion), this experiment will allow us to (1) examine a lexical influence on pseudo-word spelling by comparing the rate at which low-probability target graphemes are produced for pseudo-words with and without close lexical neighbors; (2) given the possibility that the sublexical process may be sensitive to syllabic structure or encode syllabic units (e.g., Perry et al., 2002b; Treiman et al., 2002), a comparison of the neighbor pseudo-words with the syllable controls will allow us to verify that any observed effects are lexical in origin and not attributable to target graphemes being more likely in certain syllabic contexts. In addition, the comparison of the rate of low-probability target graphemes in syllable control pseudo-words versus phoneme control pseudo-words might reveal an influence of syllabic context.

Experimental and filler pseudo-words were mixed and divided into four lists each including 3–4 neighbor pseudo-words, 3–4 phoneme control pseudo-word, 3–4 syllable control pseudo-words and 8–9 fillers. Pseudo-words derived from the same source word never occurred in the same list.

#### Procedure

Participants were asked to write down each dictated pseudo-word with the first spelling that came to mind. They were tested in groups and wrote their responses in a notebook, one pseudo-word per page. Two lists were dictated on the same day with a three hour interval; the remaining two lists were presented 1 week later under the same conditions.

### RESULTS

The data were scored by counting the number of *target graphemes* produced in each experimental condition. For each pseudo-word, the target grapheme was the low-probability, word-final spelling used in its source word. For example, for the pseudo-words /bəti/ (neighbor pseudo-word), /tãzi/ (phoneme control) and /liti/ (syllable control), all derived from “petit”/pəti/, we counted how often the final phoneme /i/ was spelled using the low-probability spelling-IT. For the participant analysis, the dependent variable was the total number of target spellings produced by each participant in each experimental condition. For the item analysis, the dependent variable was the total number of target spellings produced for each pseudo-word in each experimental condition. Because of the distribution of the data (non-normal, heterogeneity of variance), main effects were analyzed using Friedman (by participant) and Kruskal-Wallis (by items) tests while differences between paired conditions were examined using Wilcoxon Matched-Pairs Signed-Ranks Test (*Z*).

**FIGURE 2:**
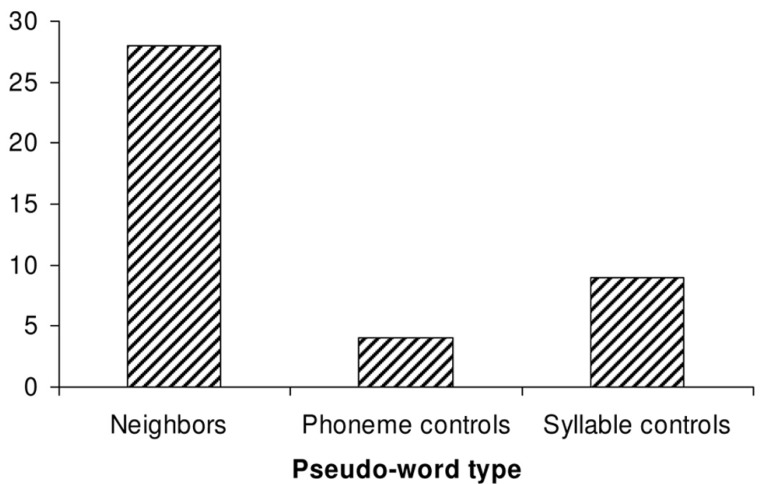
**Experiment 1: percentage of low-probability target graphemes produced in each pseudo-word spelling condition**.

Results are displayed in **Figure 2**. There was an overall main effect of pseudo-word set (by participants: χ^2^ = 33.59 (2), *p* < 0.000; by items: χ^2^ = 12.25 (2), *p* < 0.005).

More specifically, and as predicted by the hypothesis of lexical/sublexical interaction, more target spellings were produced in response to neighbor pseudo-words than to either phoneme control pseudo-words (by participants: *Z* = 4.50, *N* = 29, *p* < 0.0001; by items: *Z* = 2.94, *N* = 14, *p* < 0.005) or syllable control pseudo-words (by participants: *Z* = 4.13, *N* = 29, *p* < 0.0001; by items: *Z* = 2.63, *N* = 14, *p* < 0.01). In addition, more target spellings were produced in response to syllable controls than phoneme controls, although this difference was significant only by participants (*Z* = 2.39, *N* = 29, *p* < 0.05) but not by items (*Z* = 1.12, *N* = 14, ns).

### DISCUSSION

Experiment 1 revealed a significant lexical influence on the spelling of pseudo-words. This is reflected by the finding that low probability final graphemes were used more often when spelling pseudo-words that were close phonological neighbors of words than when spelling pseudo-words that had no such neighbor. Importantly, the results reveal that identical final syllables were spelled differently in neighbor vs. syllable control pseudo-words. This supports the prediction that the observed effect has a lexical origin and is not merely due to the target graphemes being more likely in certain syllabic contexts. There is also a trend for target spellings being more common in pseudo-words that preserve the final syllable of the source word. This effect is consistent with the view of syllables as important processing units, but should be confirmed in future studies specifically designed to address this question.

The results can be understood if we assume that upon auditory presentation of a pseudo-word, close word neighbors are activated in the phonological lexicon. This activation propagates to orthographic forms in the orthographic lexicon which, in turn, produces activation of their constituent graphemes. The sublexical process also activates a set of candidate graphemes and, as the system settles on a response, the outputs of the sublexical and lexical processes are integrated at the grapheme level (see Introduction and **Figure 1**). Thus, the higher rate of low probability target spellings in neighbor pseudo-words can be understood as deriving from the contribution of the lexicon, which is stronger when a close lexical neighbor becomes activated.

The second experiment aimed to replicate and extend these findings by testing further predictions that follow from our proposed account. If we are correct, the stronger activation of the lexical neighbor, the stronger its influence on pseudo-word spelling should be. We investigate the influence of two factors that are likely to boost this activation: (1) the degree of phonological similarity between pseudo-words and lexical neighbors (Experiment 2), using the number of shared phonetic features as a measure of phonological similarity (e.g., Connine et al., 1993) and (2) the lexical frequency of these neighbors (*post hoc* analysis combining the results of Experiments 1 and 2).

## EXPERIMENT 2: THE INFLUENCE OF PHONOLOGICAL SIMILARITY ON THE NEIGHBORHOOD EFFECT

### METHODS

#### Participants

Forty-two undergraduate students of the University Pierre Mendès-France (Grenoble) participated in this experiment in exchange for course credits. All were French native speakers, had no history of neurological disorders or dyslexia. None of them participated in Experiment 1.

#### Materials

A list of 166 pseudo-word stimuli was constructed. It included three sets of 14 experimental and 124 filler pseudo-words. As in Experiment 1, experimental pseudo-words were derived from 14 source words by substituting one or more non-final phonemes. Source words were CVCV words of medium to high frequency (mean = 177 per million, range: 4–1538; from Lexique 3, New, 2006) ending with a low probability final grapheme (e.g., “petit”/pəti/). Only three of the source words used in Experiment 2 were also used in Experiment 1. Three sets of pseudo-words were derived from the 14 source words: (1) *close phonological neighbors*: 14 pseudo-words**that differed from the source words by only one phonetic feature (10 differed in place of articulation and four in voicing; e.g., /bəti/ derived from “petit”/pəti/), (2) *distant phonological neighbors*: 14 pseudo-words that also differed from the source words by only one phoneme, but by two or three phonetic features (e.g., /vəti/ derived from “petit”/pəti/), and (3) *no neighbor*: 14 control**pseudo-words that only shared their final phoneme with the source words (/tãζi/). These stimuli allowed us to examine the effect of small differences in sub-phonemic similarity on the activation of word neighbors. Note that in Experiment 1, 10/14 pseudo-words differed from the source words by only one phonetic feature and 4/14 by two features or more.

Experimental pseudo-words and fillers were mixed and divided into two lists, each containing 21 target pseudo-words (3*7) and 62 fillers. Each list contained half of each set of pseudo-words, so that two neighbor pseudo-words derived from the same source word never occurred in the same list. At least 19 fillers occurred between two pseudo-words with an identical final phoneme.

#### Procedure

As in Experiment 1. The two lists were dictated to the same participants in two sessions one week apart. List order was counterbalanced.

### RESULTS AND DISCUSSION

**FIGURE 3:**
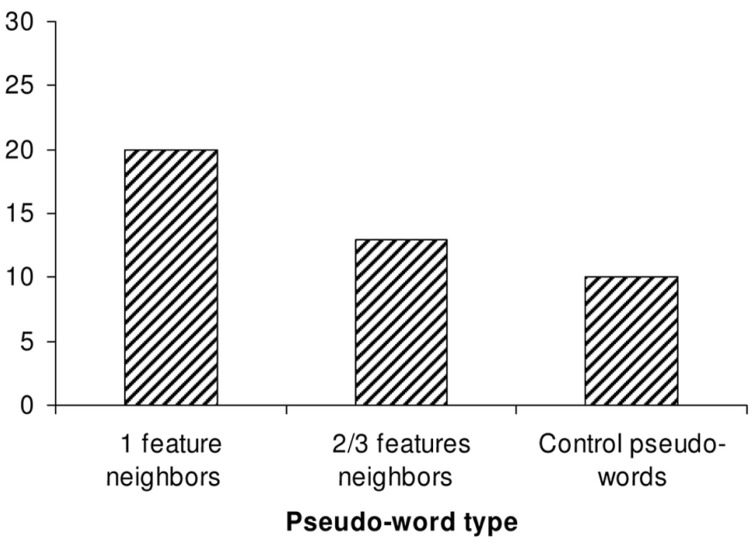
**Experiment 2: percentage of low-probability target graphemes produced in each pseudo-word spelling condition**.

The results of Experiment 2 are presented in **Figure 3**. As in Experiment 1, the data were scored by counting the number of *target graphemes* produced in each experimental condition. There was an overall main effect of pseudo-word set (by participants: χ^2^ = 23.09 (2), *p* < 0.001; by items: χ^2^ = 4.28 (2), *p* = 0.06, 1-tail). More target spellings were produced in response to neighbor pseudo-words than to control no-neighbor pseudo-words (by participants: *Z* = 4.43, *N* = 42, *p* < 0.0001; by items: *Z* = 2.04, *N* = 14, *p* < 0.05). This replicates the basic lexical neighborhood effect that was observed in Experiment 1. In addition, more target spellings were produced in response to close phonological neighbor pseudo-words (i.e., those that were only one feature away from their source word) than to more distant neighbor pseudo-words (i.e., 2–3 features away from their source word; by participants: *Z* = 3.37, *N* = 42, *p* < 0.001; by items: *Z* = 2.32, *N* = 14, *p* < 0.05). This supports our prediction that the strength of lexical neighborhood effects is modulated by the degree of phonological similarity between pseudo-words and lexical neighbors. Note that the difference in the production of target spellings between distant phonological neighbor pseudo-words and control pseudo-words only reached significance in the analysis by participants (by participants: *Z* = 1.97, *N* = 42, *p* = 0.05, by items: *Z* = 0.44, *N* = 14, ns).

In summary, Experiment 2 revealed that the lexical neighborhood effect on pseudo-word spelling is sensitive to the degree of phonological similarity between pseudo-words and lexical neighbors. This might explain why several studies (Perry et al., 2002a; Perry and Ziegler, 2004) have failed to show an influence of body neighborhood on pseudo-word spelling. Body neighbors are words that share the same orthographic rime (body), such as “spring”, “wing” and “cling”. Based on the frequency of individual phoneme-grapheme mappings, a pseudo-word such as /zaIt/ would be expected to be spelled “ZITE.” However, /zaIt/ has more body neighbors that use less common mappings (e.g., light, fright, might, bright, etc.) than body neighbors that use the more common mapping (bite, kite, rite etc.). Although this could increase the probability of the “ZIGHT” spelling for /zaIt/, this is not supported by the results of Perry and collaborators. Although body neighborhood is a variable that is related to lexical neighborhood as we have defined it, body neighbors may differ from one another by up to three phonemes (i.e., the number of phonemes that can form a syllable onset). Overall, the degree of phonological similarity between body neighbors and pseudo-word stimuli may not be sufficient to generate an observable influence on pseudo-word spelling.

## *POST HOC* ANALYSIS: THE INFLUENCE OF LEXICAL FREQUENCY ON THE NEIGHBORHOOD EFFECT

To examine the influence of lexical frequency on the magnitude of the lexical contribution to pseudo-word spelling, we measured the degree of correlation between the log frequency of the source words and the mean number of target spellings produced in response to neighbor pseudo-words. In order for the analysis to include a suitable number of items, we combined data from the neighbor pseudo-word condition of Experiment 1 and the close neighbor pseudo-word condition of Experiment 2, for a total of 28 words. The analysis revealed a significant correlation between the log^10^ lexical frequency of the source words and the number of low-probability target graphemes produced (*N* = 28, *r* = 0.52; *r*^2^ = 0.26, *t* = 2.83, *p* = 0.005), suggesting that high frequency lexical neighbors have a greater influence on pseudo-word spelling than lower frequency ones.

## GENERAL DISCUSSION

The data reported in this paper are the first to demonstrate a clear lexical influence on pseudo-word spelling in unimpaired adults in a paradigm that minimizes the likelihood of an overt recruitment of lexical processes. Our findings offer direct evidence that strengthen earlier empirical reports of lexical priming effects on pseudo-word spelling, extend results obtained with children, and challenge the view that lexical and sublexical spelling processes are strictly independent.

To account for the lexical neighborhood effect observed in this study, we have proposed that, upon hearing pseudo-words, close neighbors are activated in the phonological lexicon. This in turn activates the spellings of these word neighbors. The notion that spoken stimuli (words or pseudo-words) activate the representations of their phonological neighbors is well accepted in the spoken word recognition literature (for a review see: Gow, 2012). Consistent with the results of Experiment 2, Connine et al. (1993) have shown that pseudo-words can prime phonological word neighbors as long as the phonological distance between the two does not exceed one or two phonetic features (see also: Milberg et al., 1988; Marslen-Wilson et al., 1996; Frisch et al., 2000; Bölte and Coenen, 2002; Saito et al., 2003; Raettig and Kotz, 2008; Gow, 2012). The claim that lexical orthography may be automatically activated during spoken word processing is also supported by a variety of studies (e.g., Whatmough et al., 1999; Ziegler et al., 2003; Ventura et al., 2004). In this study, we have extended the implications of these claims to the context of spelling to dictation, and have adopted the further assumption that both lexical and sublexical processes, operating in parallel, activate a common set of graphemes (see also: Tainturier and Rapp, 2001, 2004; Rapp et al., 2002; Bosse et al., 2003; Bonin and Delattre, 2010; Purcell et al., 2011; Martin and Barry, 2012; Roux et al., 2013). The sublexical system activates various graphemic units as a function of the relative frequency/probability of the phonology to orthography mappings in the language and these compete for selection with one another as well as with the graphemes activated by the lexical system. If, as we have observed, a pseudo-word has a close lexical neighbor with a low probability phoneme-grapheme correspondence then this grapheme is more likely to be a successful competitor than in the case of a pseudo-word that has no such neighbor. This supports our proposal that, as information from these two processes is integrated, the build-up of lexical activation is sufficient to exert an influence on pseudo-word spelling (for a discussion of the computational purposes served by lexical-sublexical integration at this level, see: Folk et al., 2002; Houghton and Zorzi, 2003).

Our proposal assumes that the lexical influence affects the selection of abstract graphemes for production. However, as indicated in the Section “Introduction,” past studies have suggested that the lexical priming may be understood as the result of lexically driven modification of the relative weights of competing sublexical phoneme-grapheme mappings (Perry, 2003). While this may indeed take place under certain experimental conditions and contribute to the learning and updating of phoneme-grapheme mapping frequencies, it is unlikely to have played a significant role in the results we have reported. This is because, in the paradigm we have adopted, no word stimuli are presented and, therefore, it is unclear how a lexically generated outcome would have modified the sublexical weightings of phoneme-grapheme options prior to the sublexical processing of the pseudo-word stimulus. In fact, our proposal can account for both the findings we report as well as at least some of the lexical priming effects that have been observed in previous paradigms without positing a re-weighting mechanism. This is because the presentation of a closely related pseudo-word target stimulus following the presentation of a word prime would lead to the re-activation of the spelling of the word prime. This would increase the likelihood that the pseudo-word would be spelled by analogy to the prime without a need to posit a prior re-weighting of phoneme-grapheme mappings as the source of the priming effect. Hence, the paradigm we have used is a more powerful way of measuring lexical influence on pseudo-word spelling than is the priming paradigm. In sum, while it is likely that a re-weighting of phoneme-grapheme correspondences occurs under certain circumstances, it is unlikely to account for the results we have reported here and it is certainly not required to explain previous results.

It is quite possible that the results we have reported here could be accommodated within other architectures of the spelling process, including frameworks that do not assume an explicit lexical-sublexical distinction (e.g., Graham et al., 1997, 2000; Patterson and Lambon Ralph, 1999). However, it is beyond the scope of this paper to discuss this issue in detail and we refer the interested reader to the discussions of this question by Bosse et al. (2003), Folk and colleagues (Folk et al., 2002; Folk and Rapp, 2004; Jones et al., 2009) and a lively debate by Rapcsak et al. (2007) in support of the dual process explanation.

In summary, although the details of lexical-sublexical integration remain to be specified (but see Houghton and Zorzi, 2003, for a possible computational implementation) our results clearly indicate that the phonological similarity between a pseudo-word and its lexical neighbor as well as the neighbor’s lexical frequency affect the strength of the lexical contribution to the spelling of the pseudo-word. Thus, these results provide constraints on our understanding of the processes that interact in producing spellings for both familiar and unfamiliar words.

## Conflict of Interest Statement

The authors declare that the research was conducted in the absence of any commercial or financial relationships that could be construed as a potential conflict of interest.
